# Supplementary material for: Cellular Transcriptomics of Carboplatin Resistance in a Metastatic Canine Osteosarcoma Cell Line
Source: Genes (Basel). 2023 Feb 23;14(3):558. doi: 10.3390/genes14030558 (PMC10048144; doi:10.3390/genes14030558)
Supplement: Supplementary file 1 [file genes-14-00558-s001.zip › genes-HMPOS_supplement_F.pdf]

Supplementary Figures

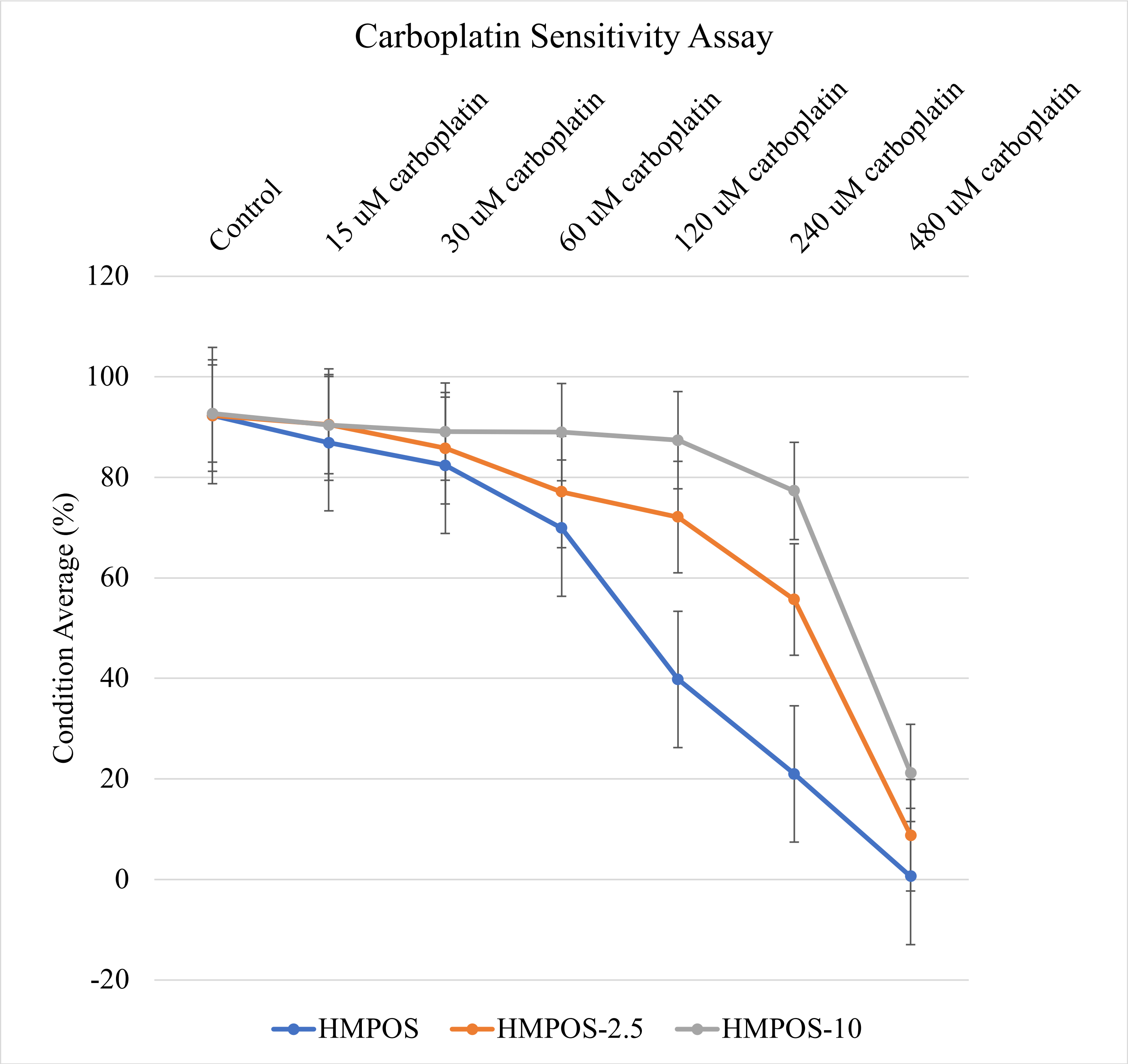

Supplementary Figure S1. Condition averages of triplicates for each HMPOS cell line ate each carboplatin dosage with standard error bars.

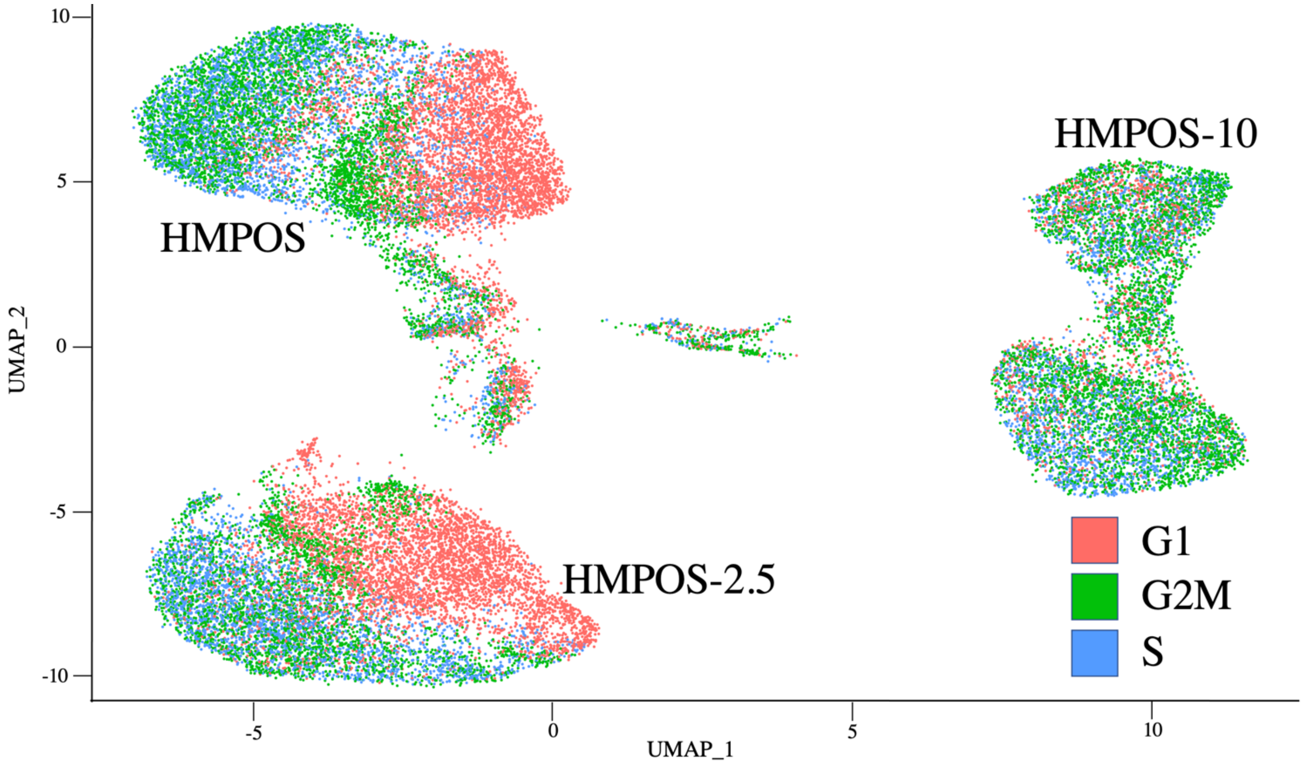

Supplementary Figure S2. A UMAP of the distribution of cell phases for the three HMPOS cell lines. The scRNAseq data underwent cell cycle scoring and regression before further analysis. Note the significant drop in identifiable G1 phase cells in the HMPOS-10 which indicates more rapid proliferation.

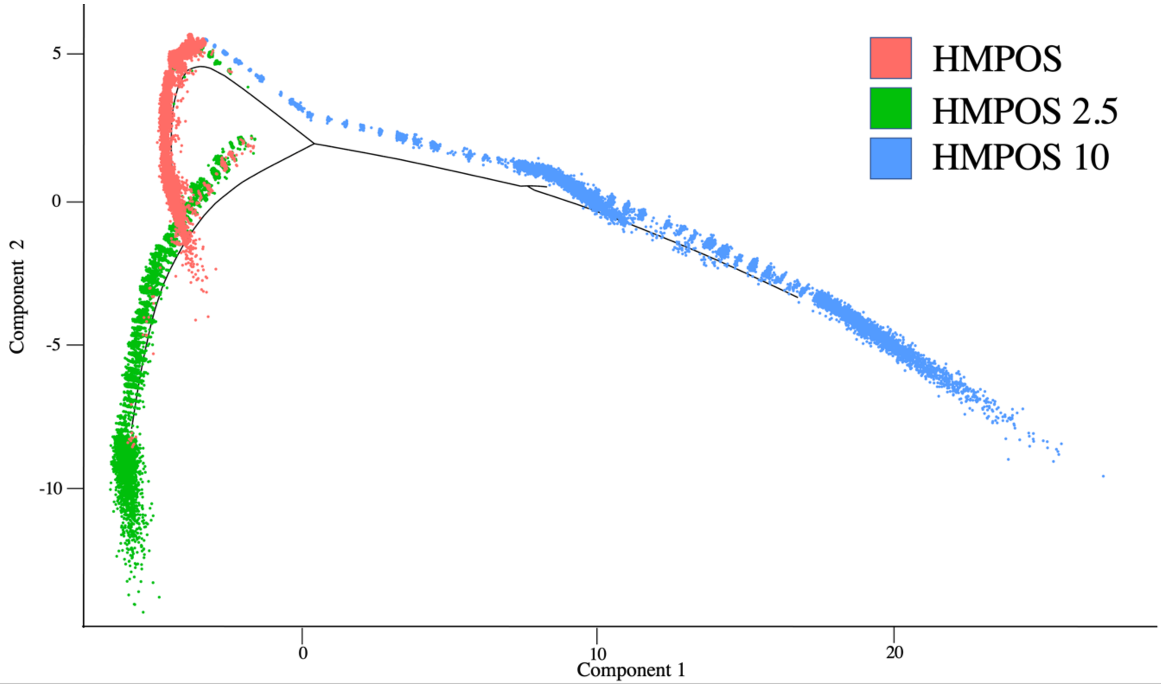

Supplementary Figure S3. A monocle pseudotime trajectory plot showing the progress of each cell in differentiating from a beginning state to a terminal state. HMPOS and HMPOS-2.5 have overlap and have a shorter trajectory indicating less differentiation and evolution. HMPOS-10 has a longer trajectory and a small branch which indicates HMPOS-10 cells are progressed further in evolving under pressure of carboplatin exposure.

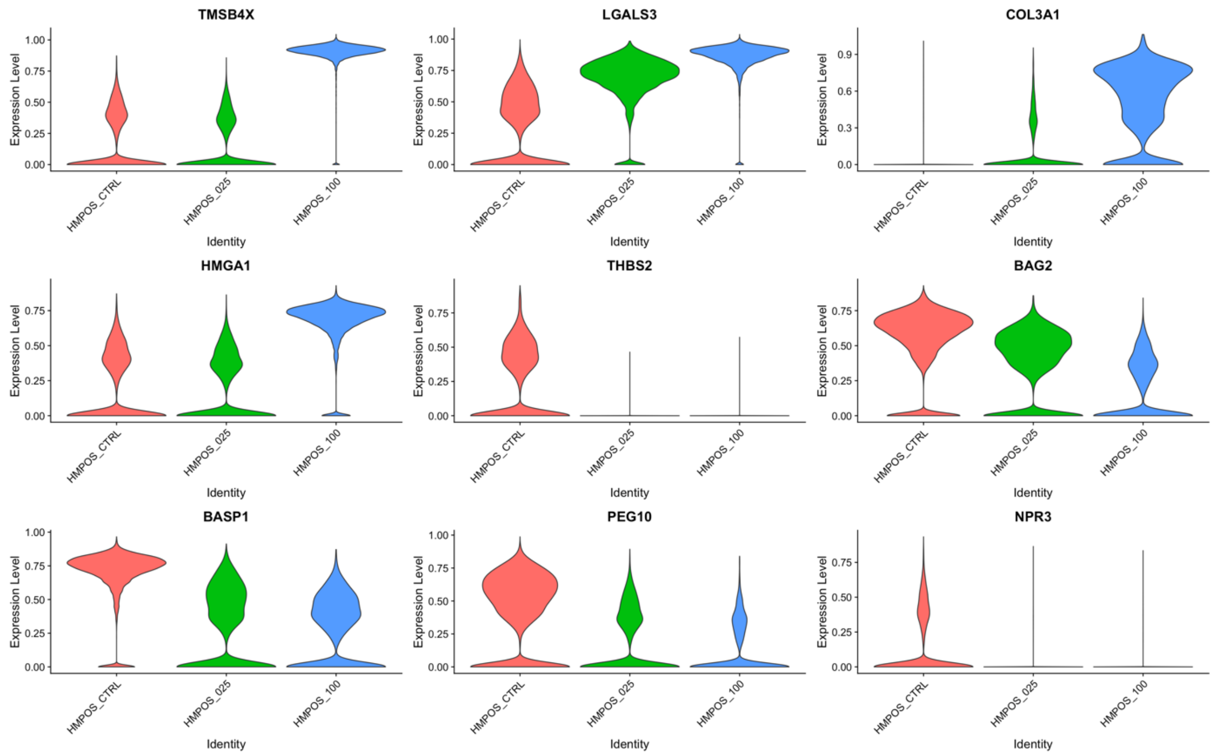

Supplementary Figure S4. Violin plots of gene expression of genes that showed an increase with an increase or decrease in expression in correlation with the increase in chemotherapy respectively. *TMSB4X*, *LGALS3, COL3A1*, and *HMGA1* were upregulated as chemotherapy resistance increased. These genes are related to chemoresistance and EMT [32-47]. *THBS2, BASP1*, and *NPR3* were downregulated as chemotherapy resistance increased and downregulation or knockout of these genes is associated with chemoresistance and EMT [48-50]

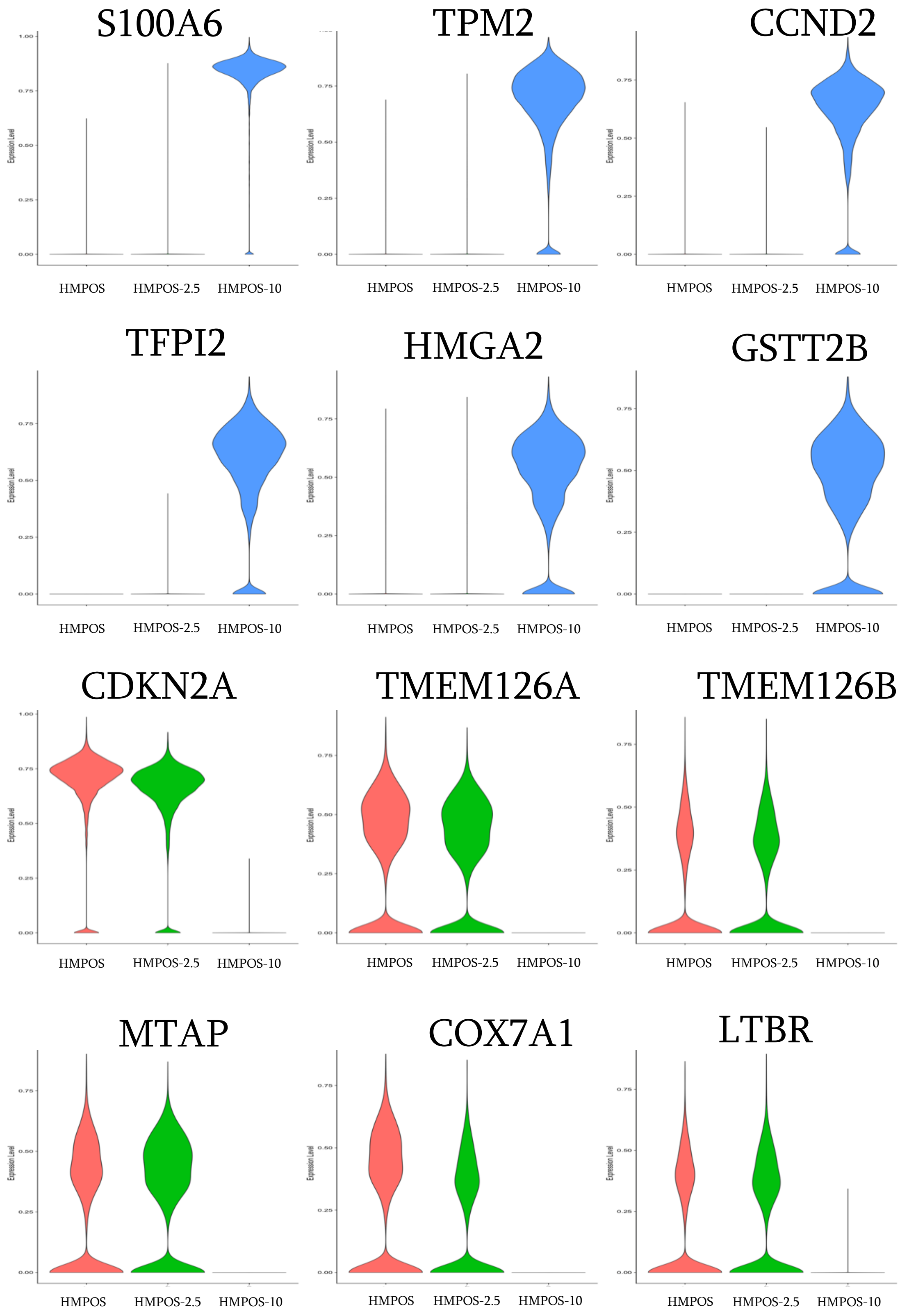

Supplementary Figure S5. Violin plots of expression of genes that were highly upregulated or downregulated in HMPOS 10. *S100A6*, *TPM2*, *CCND2*, *TFPI2*, *HMGA2,* and *GSTT2B* were upregulated in HMPOS-10 and are associated with chemotherapy resistance and EMT [60-74]. *CDKN2A*, *TMEM126A*, *TMEM126B, MTAP*, *COX7A1*, and *LTBR* were downregulated in HMPOS-10 and downregulation of these genes promotes cancer development and EMT [75-87].

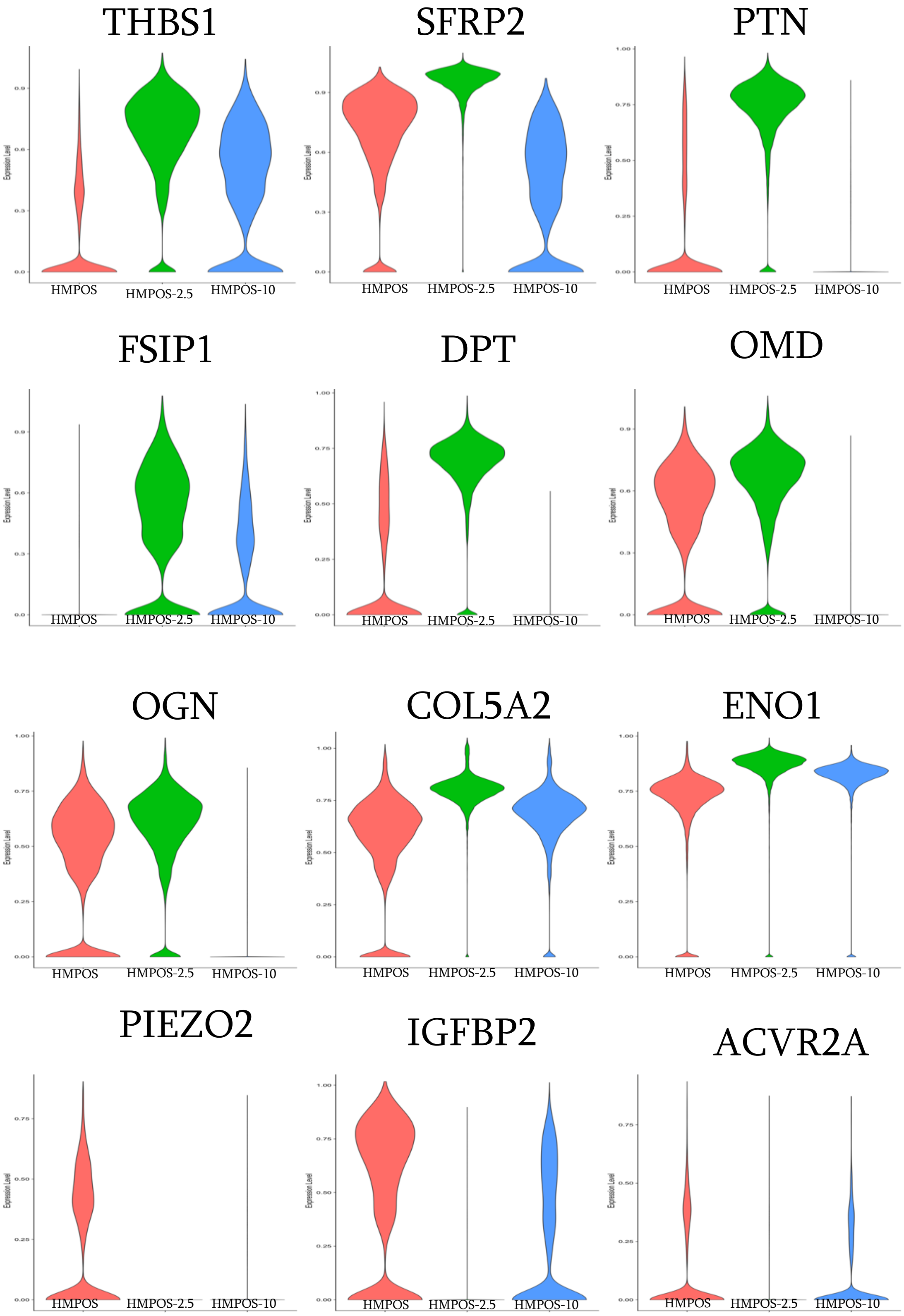

Supplementary Figure S6. Violin plots of gene expression of genes that were highly upregulated or downregulated for HMPOS 2.5. *THBS1*, *SFRP2*, *PTN*, *FSIP1*, *DPT*, *OMD*, *OGN*, *COL5A2*, and *ENO1* were upregulated and associated with promoting EMT and metastasis [88-107]. *PIEZO2*, *IGFBP2*, and *ACVR2A* were downregulated and these genes are associated to promote EMT with upregulated [108-110].

A

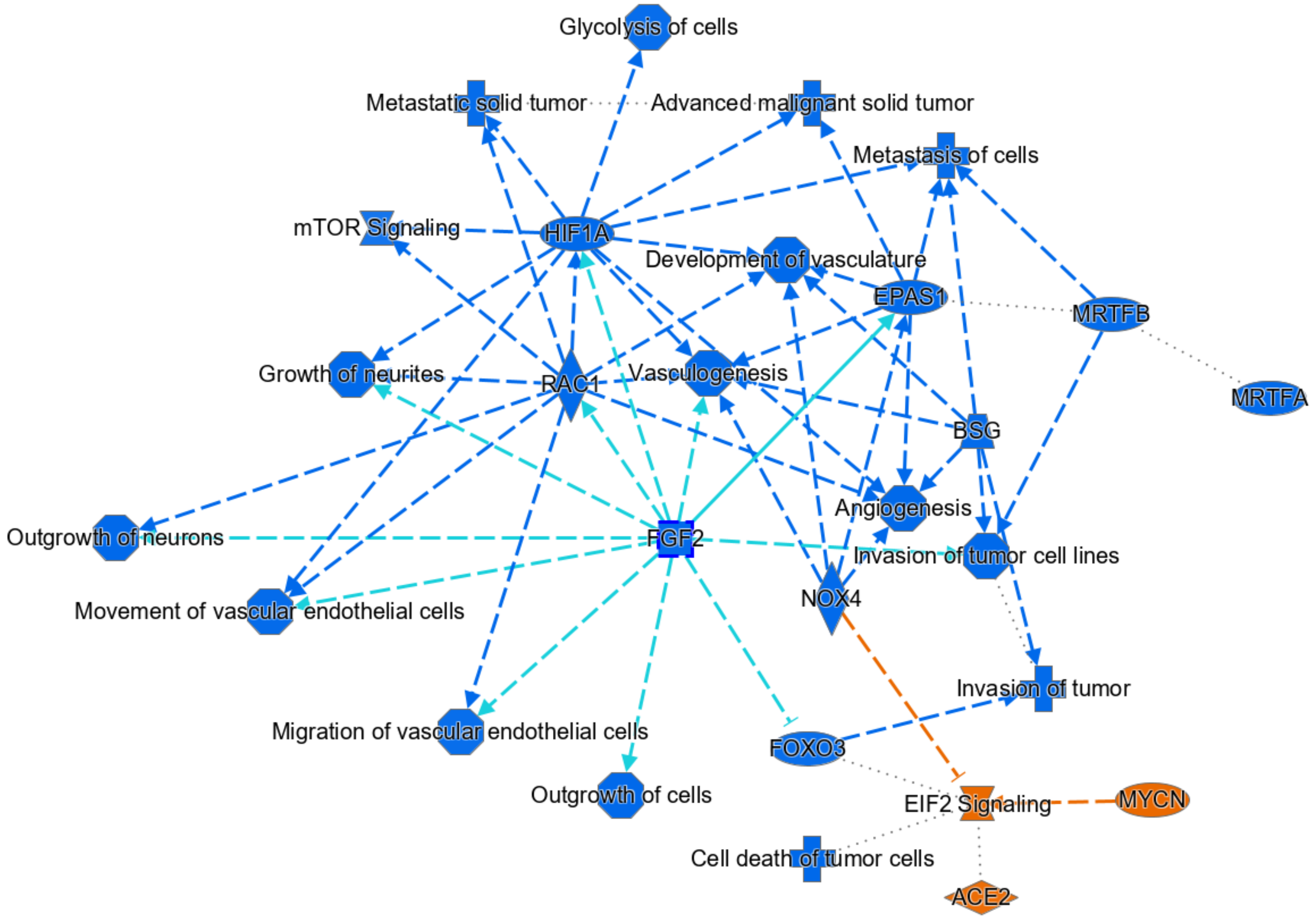

B

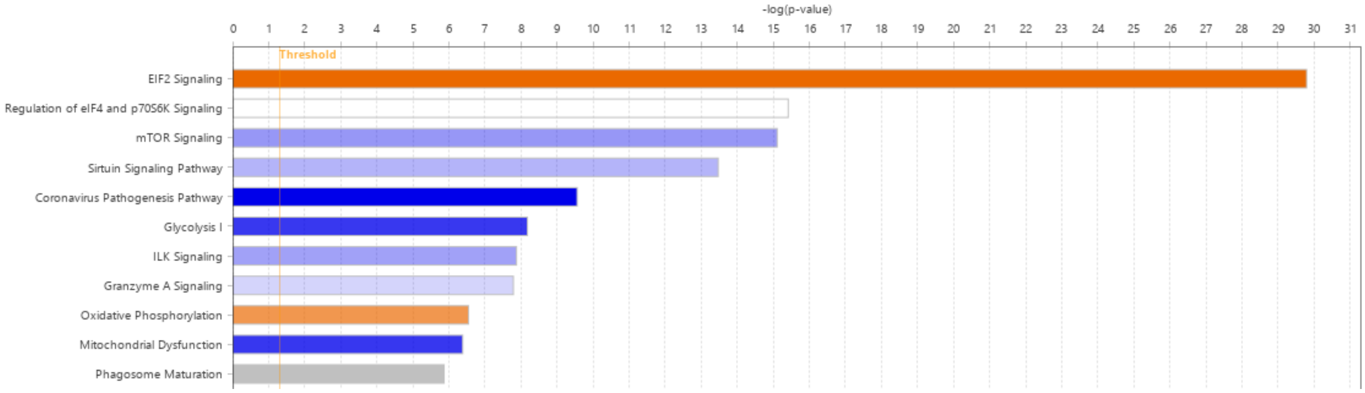

Supplementary Figure S7. A) Network representation of the core analysis results for the differential expression gene list comparing **HMPOS to HMPOS-2.5**. Each node represents a pathway, regulator, gene, or function significantly misregualted in the comparison (Fisher's exact p <0.05). Orange denotes activation and blue denotes inhibition. B) Pathway analysis for the same list. Orange denotes a positive z-score and blue a negative z-score. Downregulation of HIF1A and mTOR signaling is observed, along with a decrease in cell migration.

A

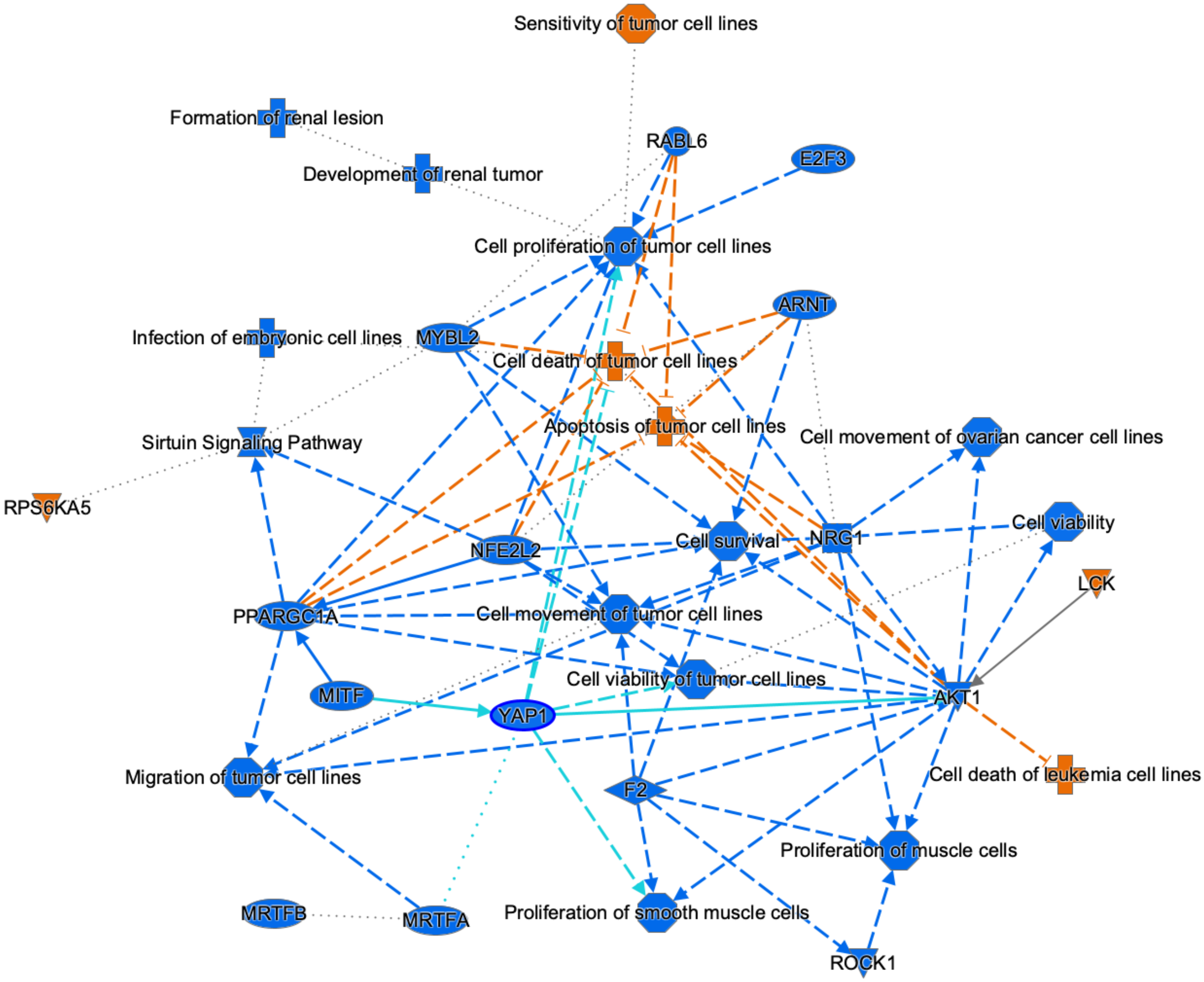

B

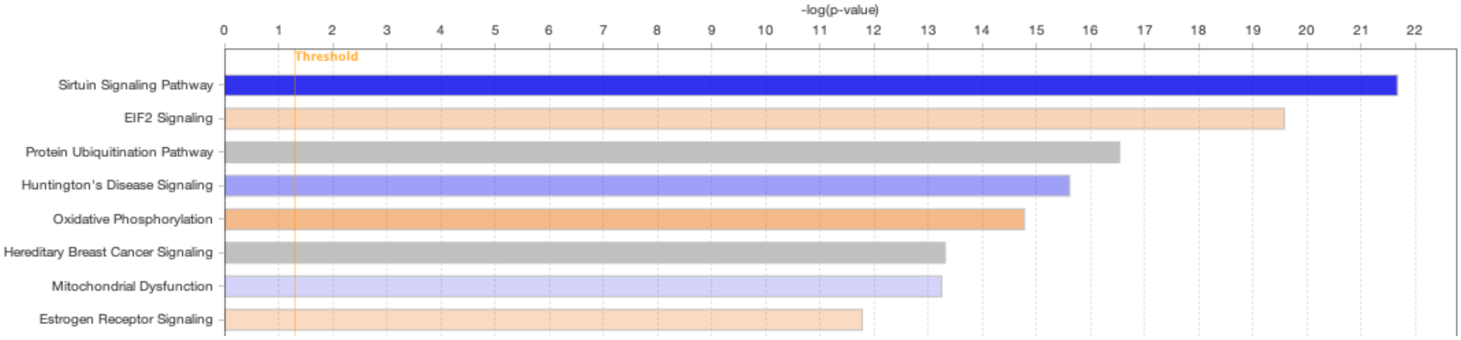

Supplementary Figure S8. A) Network representation of the core analysis results for the differential expression gene list comparing **HMPOS to HMPOS-10**. Each node represents a pathway, regulator, gene, or function significantly misregualted in the comparison (Fisher's exact p <0.05). Orange denotes activation and blue denotes inhibition. B) Pathway analysis for the same list. Orange denotes a positive z-score and blue a negative z-score.

A

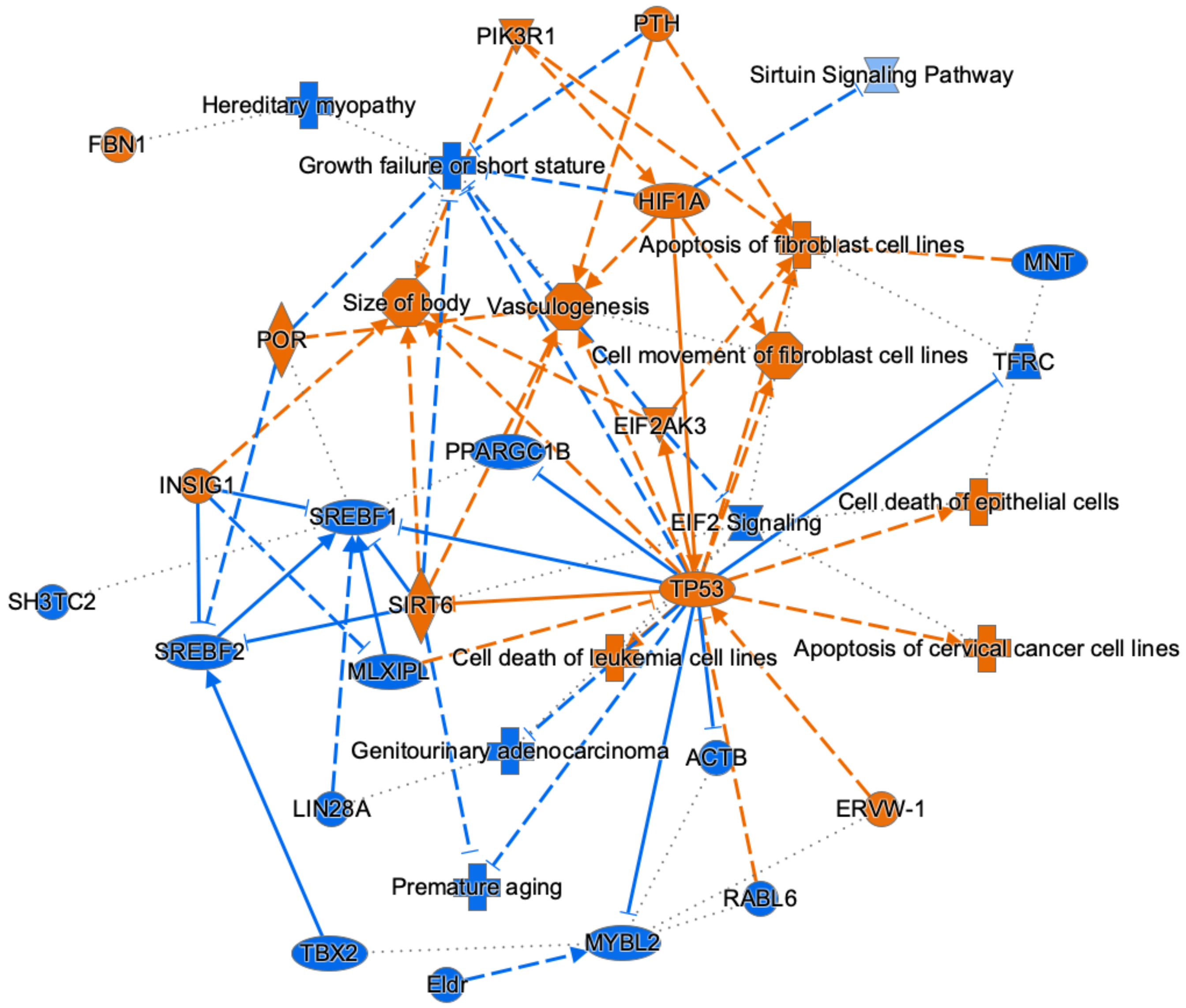

B

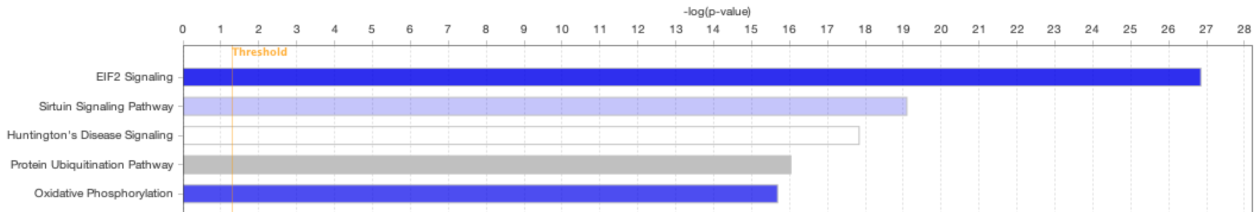

Supplementary Figure S9. A) Network representation of the core analysis results for the differential expression gene list comparing **HMPOS-2.5 to HMPOS-10**. Each node represents a pathway, regulator, gene, or function significantly misregualted in the comparison (Fisher's exact p <0.05). Orange denotes activation and blue denotes inhibition. B) Pathway analysis for the same list. Orange denotes a positive z-score and blue a negative z-score. Stimulation of *TP53* and *HIF1A* with an inhibition of the sirtuin pathway is seen in HMPOS-2.5.

A

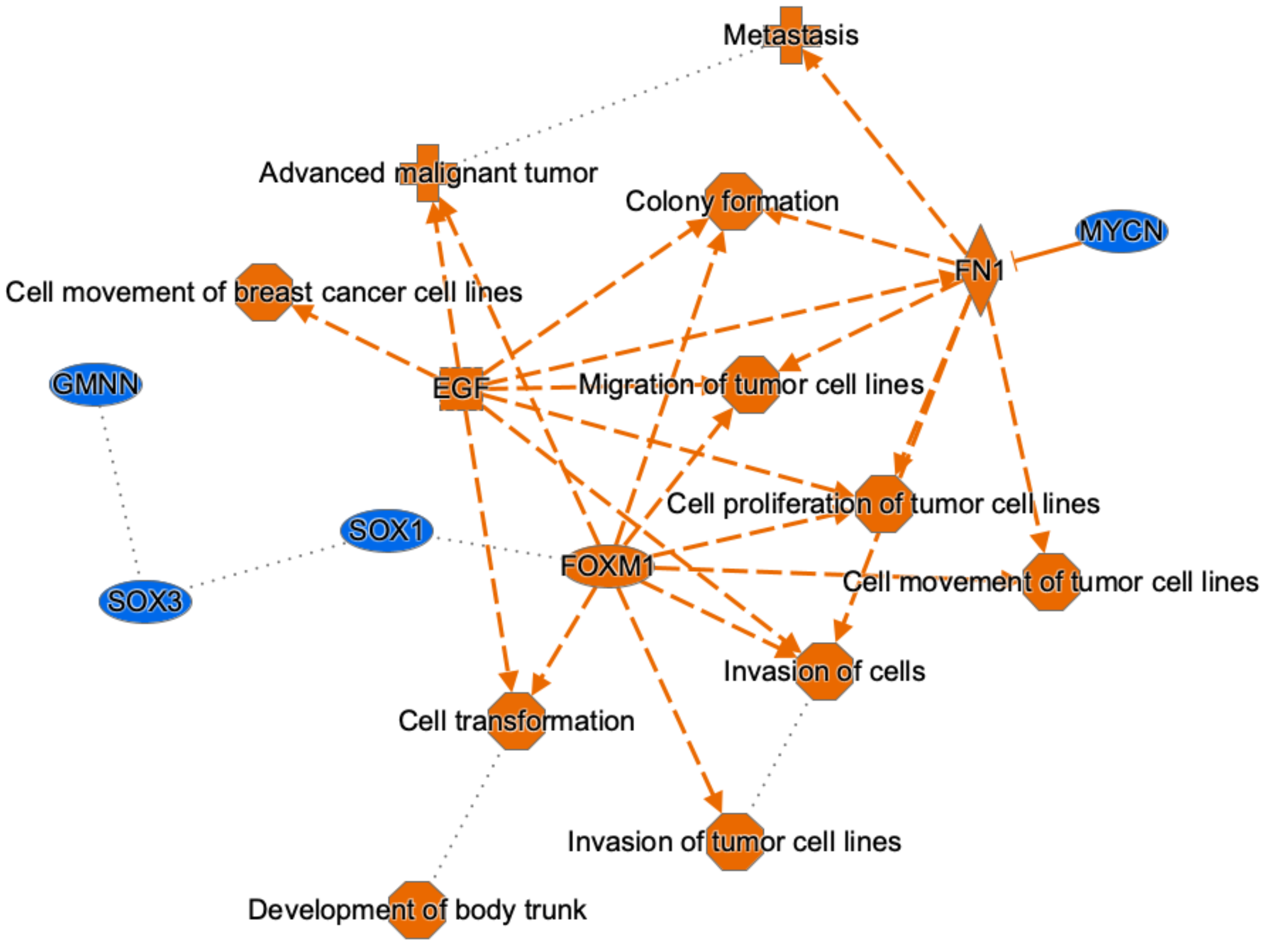

B

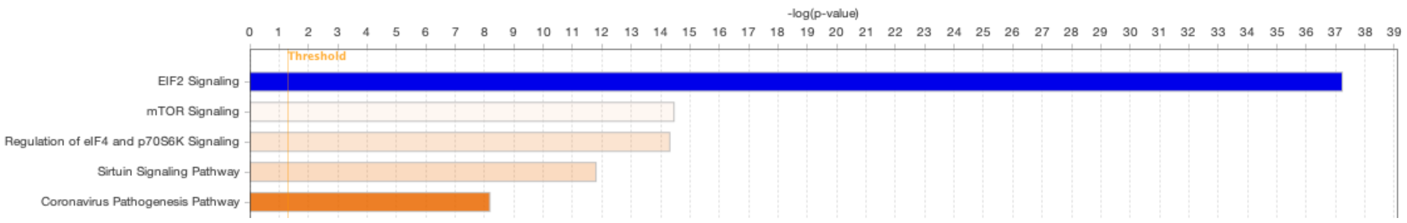

Supplementary Figure S10. A) Network representation of the core analysis results for the differential expression gene list comparing **HMPOS to HMPOS-Var1**. Each node represents a pathway, regulator, gene, or function significantly misregualted in the comparison (Fisher's exact p <0.05). Orange denotes activation and blue denotes inhibition. B) Pathway analysis for the same list. Orange denotes a positive z-score and blue a negative z-score.

A

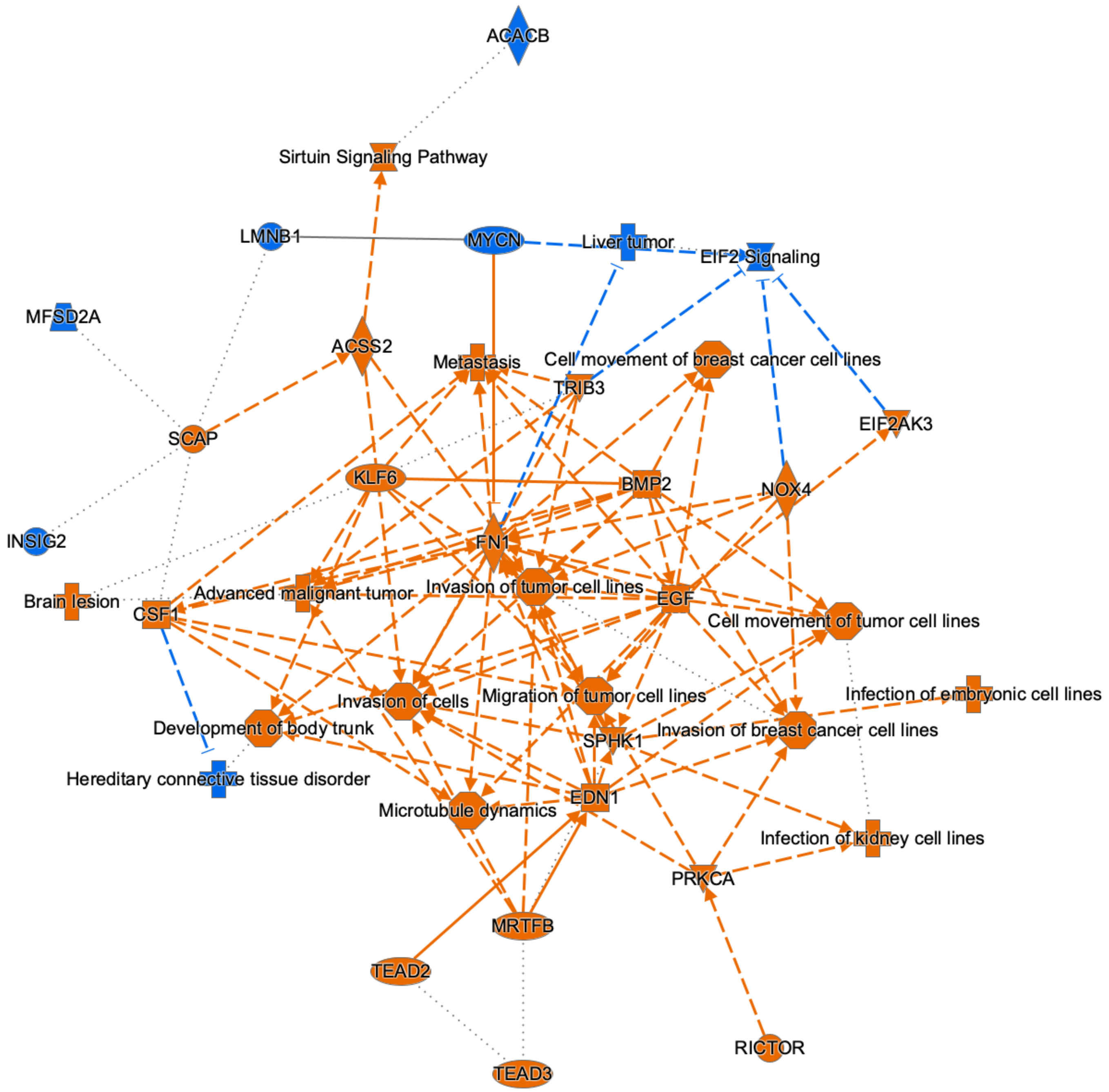

B

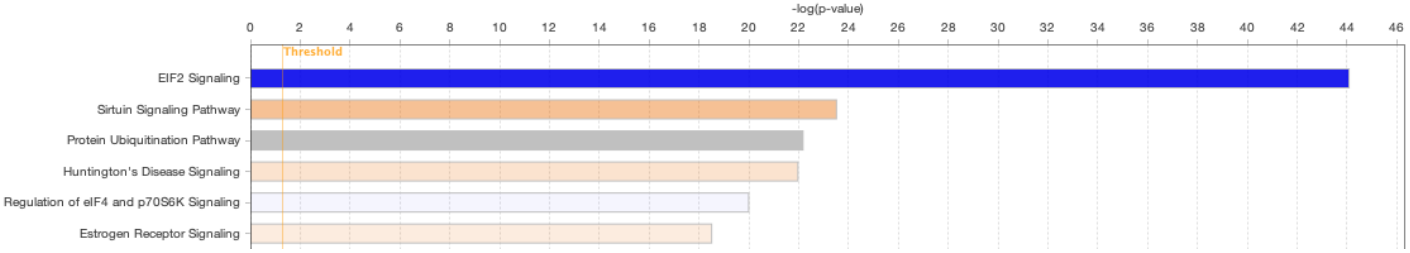

Supplementary Figure S11. A) Network representation of the core analysis results for the differential expression gene list comparing **HMPOS-2.5 to HMPOS-2.5-Var**. Each node represents a pathway, regulator, gene, or function significantly misregualted in the comparison (Fisher's exact $p < 0.05$). Orange denotes activation and blue denotes inhibition. B) Pathway analysis for the same list. Orange denotes a positive z-score and blue a negative z-score.

A

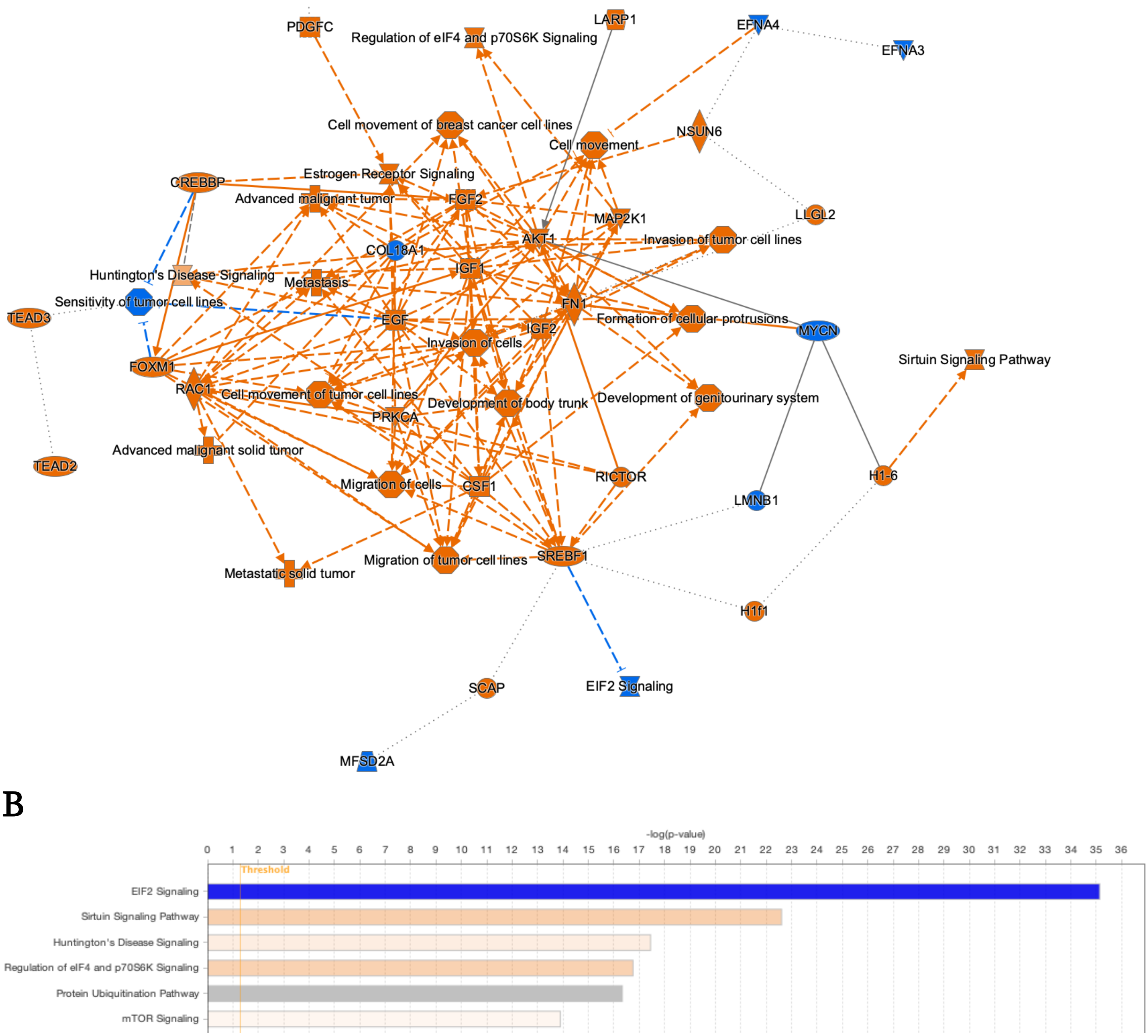

B

Supplementary Figure S12. A) Network representation of the core analysis results for the differential expression gene list comparing **HMPOS-10 to HMPOS-10-Var**. Each node represents a pathway, regulator, gene, or function significantly misregualted in the comparison (Fisher's exact p <0.05). Orange denotes activation and blue denotes inhibition. B) Pathway analysis for the same list. Orange denotes a positive z-score and blue a negative z-score. Considerable upregulation associated with IGF1 and metastatic cell movement is observed, similar to the comparisons of HMPOS-10 to other cells.

A

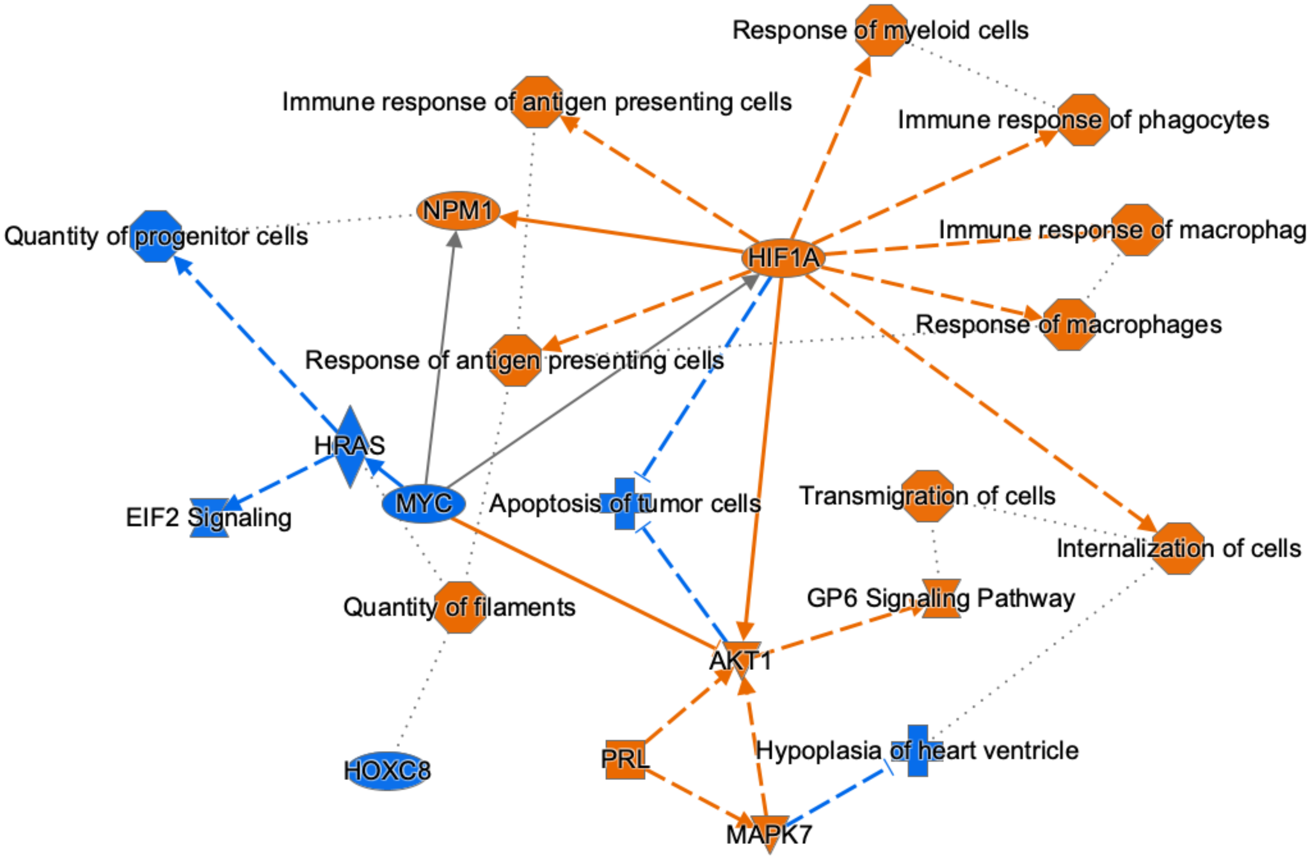

B

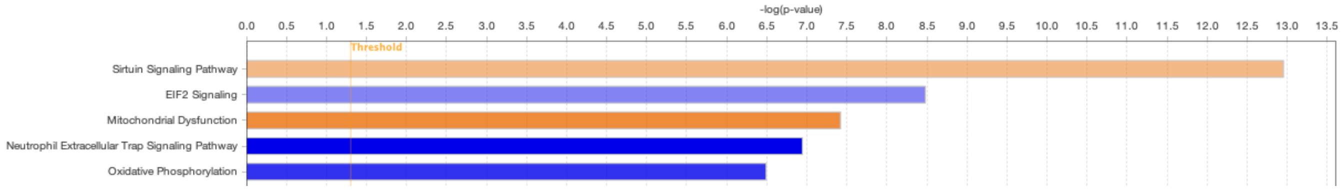

Supplementary Figure S13. A) Network representation of the core analysis results for the differential expression gene list comparing the subclusters within **HMPOS-10 (clusters 5 to 1)**. Each node represents a pathway, regulator, gene, or function significantly misregualted in the comparison (Fisher's exact p <0.05). Orange denotes activation and blue denotes inhibition. B) Pathway analysis for the same list. Orange denotes a positive z-score and blue a negative z-score. Upregulation of *HIF1A* concomitant with stimulation of the sirtuin pathway and inhibition of EIF2 signaling is observed.
